# New Method to Implement and Analysis of Medical System in Real Time

**DOI:** 10.3390/healthcare10071357

**Published:** 2022-07-21

**Authors:** Yahia Zakria Abd Elgawad, Mohamed I. Youssef, Tarek Mahmoud Nasser, Amir Almslmany, Ahmed S. I. Amar, Abdelrhman Adel Mohamed, Naser Ojaroudi Parchin, Raed A. Abd-Alhameed, Heba G. Mohamed, Karim H. Moussa

**Affiliations:** 1 College of Engineering, Al_Azhar University, Cairo 11651, Egypt; yahia_elrefaay@hotmail.com (Y.Z.A.E.); mohiyosof@gmail.com (M.I.Y.); tarekmahmoudn1967@hotmail.com (T.M.N.); 2 Dept. of Electronics & Comm, Air Defence College, Alexandria University, Alexandria 21526, Egypt; dr.mslmany.7256.adc@alexu.edu.eg (A.A.); ahmed.s.i.amar.adc@alexu.edu.eg (A.S.I.A.); abdou40105@gmail.com (A.A.M.); 3 School of Engineering and the Built Environment, Edinburgh Napier University, Edinburgh EH10 5DT, UK; n.ojaroudiparchin@napier.ac.uk; 4 Faculty of Engineering and Informatics, University of Bradford, Bradford BD7 1DP, UK; r.a.a.abd@bradford.ac.uk; 5 Department of Electrical Engineering, College of Engineering, Princess Nourah bint Abdulrahman University, P.O. Box 84428, Riyadh 11671, Saudi Arabia; 6 School of Internet of Things, Xi’an Jiaotong-Liverpool University, Suzhou 215123, China; karim.moussa@xjtlu.edu.cn

**Keywords:** COVID-19, fuzzy logic, GSR, heart rate, IoT, machine learning, medical devices, microcontrollers

## Abstract

The use of information technology and technological medical devices has contributed significantly to the transformation of healthcare. Despite that, many problems have arisen in diagnosing or predicting diseases, either as a result of human errors or lack of accuracy of measurements. Therefore, this paper aims to provide an integrated health monitoring system to measure vital parameters and diagnose or predict disease. Through this work, the percentage of various gases in the blood through breathing is determined, vital parameters are measured and their effect on feelings is analyzed. A supervised learning model is configured to predict and diagnose based on biometric measurements. All results were compared with the results of the Omron device as a reference device. The results proved that the proposed design overcame many problems as it contributed to expanding the database of vital parameters and providing analysis on the effect of emotions on vital indicators. The accuracy of the measurements also reached 98.8% and the accuracy of diagnosing COVID-19 was 64%. The work also presents a user interface model for clinicians as well as for smartphones using the Internet of things.

## 1. Introduction

It has become necessary to combine information technology and medical discoveries to improve the quality of medical services provided to patients as a result of advancements in both. Mohamed Bakry El_Mashade et al. [1] presented a simple design for a device that takes biometrics. They analyzed the biometrics and their behavior using a fuzzy logic model. Three different types of sensors connected to the microcontroller were used to transmit data. The researchers tested the proposed device on four hundred people and compared the results with similar devices available in the local market. Despite all this effort, noise-canceling circuits were not used, and they relied only on the use of amplifiers to amplify the received signal and microcontrollers to convert from analog to digital.

Abdulrahman K. Eesee focused on three variables to provide statistical analysis to examine people’s feelings. The GSR was used to monitor people’s feelings and check the reliability of the readings. The results were taken by displaying groups of images to monitor feelings (sad, happy, neutral). This paper confirmed that using images only as a trigger for emotion monitoring was not a satisfactory result [2]. S. Karanchery and S. Palaniswamy found a way to help people with autism spectrum disorders communicate their emotions to healthcare providers. The participants were shown a set of 125 images to monitor and display their emotions. The readings were then entered into a database for analysis, and the proposed methodology’s accuracy in this study reached 99.8% [3]. H. F. Azgomi et al. established a simulation of a cognitive stress state using the skin conduction and closed-loop method. The researchers relied on data collection and modeling of skin behavior response events to external stimuli. The authors emphasized that the proposed simulation is the initial step for treating cognitive disorders using the brain-state decoding method [4].

F. A. Satti et al. conducted a study and analysis of the impact of digital games and the emergence of violence on players. They used galvanic skin response (GSR) to collect player data during a game session to monitor changes in mood. A model was built indirectly based on the use of more than one machine learning model to determine the stress a player was experiencing. This work confirmed that the proposed model achieved a high accuracy rate compared to similar models, which amounted to 63.39% [5]. I. K. Hanoon and M. I. Aal-Nouman designed a device to help patients check their health status, send medical data to doctors and display data with the Internet of things platform. The researchers used sensors to measure three important vital parameters (heart rate, oxygen level, temperature). Arduino was primarily used in the proposed design to transfer data from sensors to the cloud and then to doctors [6].

Several studies have been submitted that use the LabVIEW(NI, LabVIEW NXG 5.1 Austin, Texas, U.S.) tool to compare heart rate, ECG data and temperature to identify potential diseases. Some of these proposed designs were based on a DAQ and an improved method for processing signals in the time and frequency domains, whereas other systems relied on the Arduino, the myRIO module, or the PIC16f877 microcontroller. The ESP8266 WiFi module was used to transmit data to the ThingSpeak server. The data were collected for use in prediction and diagnostic models based on a machine learning algorithm. Some systems had unique features, such as requiring the doctor to issue voice commands to the monitoring system so that the system can begin taking measurements automatically. All of these studies concluded that the proposed systems were extremely effective at detecting heart disease early on [7,8,9,10].

Since 2018 there has been interest in developing new methods for detecting emotions using GSR. Researchers developed a tabulation of negative and positive effects to obtain good data for use in machine learning. The machine learning model used in this development relied heavily on the vector machine algorithm. This proposed design had an accuracy of 75.65%. The development did not end there, as in 2019 a new system was proposed using deep learning with a set of data taken for the electrocardiogram and galvanic skin response. The deep learning model was designed to identify and categorize emotions. This model was based on a pre-existing AMIGOS dataset. Despite the effort, the researchers did not address the effect of positive and negative emotions on these measures. Previous research also did not provide a database of more than two or three types of feelings. Additionally, the proposed systems were not tested in previous research on more than 100 people [11,12]. The importance of using the Internet of things to transmit medical data remotely has recently emerged, particularly at the start of 2020. Many studies have suggested and offered health monitoring systems that use the Internet of things to illustrate data to doctors and hospitals. Health monitoring systems are designed based on sensors to measure the vital parameters of patients (heart rate, body temperature, cardiac activity, peripheral capillary oxygen saturation). To process data and send data to the cloud via WiFi, the sensors were connected to a Raspberry Pi, Arduino Uno, or NodeMCU as the controller. If vital signs reach critical levels, the proposed systems send alerts to doctors or patients’ relatives. Some of this research concerns the method of encrypting data that is sent to the cloud as an important development to protect privacy. All of these data were collected and used to train a prototype machine learning model to predict what type of disease a patient might have. The Internet of things (IoT) system has enabled the development of an intelligent structure that enables physicians to monitor a patient’s health status in real time [13,14,15,16,17,18].

Health monitoring systems have also been developed to be smarter by sending alerts of a patient’s condition via text messages or determining their current location using GPS. Like its predecessor, most of the design was based on ARDUINO or NodeMCU as the microcontroller for processing measured data from sensors. Recently, researchers have been interested in developing machine learning models using data measured by these systems to predict and diagnose diseases early, such as COVID. Due to the use of IoT technology, these systems addressed the problem of patients in remote areas being unable to reach doctors [18,19,20,21]. Although the efforts made in the field of designing health and emotional-state monitoring systems are large, multiple, and highly accurate, most previous research did not use any more than three measurement sensors. Most of them resorted to the determination of human emotions using an ECG, EEG and GSR. Not all of these indicators were practical to measure in real life or to create a portable device for personal use. The researchers also did not use large databases. It was in their view that large databases reduce the efficiency of machine learning models. Researchers have not given much attention to the influence of different human emotions on vital parameters despite their importance.

Jeon et al. [22] presented a study analyzing survival rates for emergency patients in mobile emergency units inside ambulances. This study revealed that emergency external pacemakers must be accurate and easy to use. Disease-monitoring equipment must also be quick to respond. This study also proved that traffic congestion and insufficient training of the workforce are important factors in the survival of patients until reaching the hospital. Sakphrom et al. [23] designed a wristwatch based on the ESP32 and three sensors to monitor vital signs with the Internet of things platform. The measured data were processed and displayed on the wristwatch, as well as sent to the medical staff in the event of a critical condition. The proposed design is characterized by its low cost and can be used for bedridden patients or patients with mild symptoms of COVID. The proposed system was tested on a sample of 60 people, and it achieved an acceptable level of error rate of 1.22% for blood pressure and 0.13% for temperature, according to the comparison with similar devices. The time delay was 10 s, which is caused by the distance between the wristwatch and the router. Hedderich et al. [24] presented a course for medical professionals to explain the importance and effectiveness of artificial intelligence in the medical fields for predicting and diagnosing various diseases. The course dealt with a detailed explanation of the concept of artificial intelligence and provided many illustrative examples of the use of artificial intelligence in various medical fields. The course emphasized that artificial intelligence leads to better diagnosis and prediction of clinical schedules and incurable diseases. Garces et al. [25] used cloud services and machine learning technology to provide effective tools for doctors to monitor and diagnose diseases in remote areas with low cost and high accuracy. In their study, vital signs were taken from residents of a specific area daily for seven days and transferred to a database for analysis. Classifiers were used in machine learning technology to identify the patterns of the collected data. These data were presented to doctors and their diagnosis was presented and recorded within the machine learning system to train it to then perform the prediction and automated diagnosis process. The environment of the Internet of things provides easy and simple access to the data environment and provides ease of handling by doctors. Zachos et al. [26] proposed a system based on the Internet of things to detect AIDS. This was achieved by collecting data using the Internet of things system dedicated to detecting AIDS and measuring minute deviations in that data. The proposed system relied on machine learning technology in order to detect anomalies in the data that were entered into the system. A set of six machine learning algorithms was tested to reach the highest possible accuracy in data classification.

The aim of this work is to provide an integrated system using hardware and software that monitors health conditions and predicts with the possibility of diagnosing diseases by using the integrated system called EGYXOS, which is divided into three main parts (measuring the proportion of gases in the blood, measuring and analyzing vital parameters to study their impact on feelings and using those measurements in the prediction and diagnosis of some diseases). In this work, human vital parameters are measured using nine biosensors based on microcontrollers. Gas concentrations in the blood are measured through breathing and using five sensors; thus, each of them measures a specific concentration of only one type of gas. Heart rate, blood pressure, temperature and galvanic skin response are measured and recorded in a database for analysis. Practical testing of the proposed system is conducted on more than 500 people between the ages of 3 and 72 years. By collecting all this data, a large database of health parameters is created which can be used to train the advanced machine learning model. The proposed machine learning model relies on more than one algorithm to work in order to obtain the highest accuracy in analysis and prediction. The second section of this paper presents the methodology and the functional structure of the proposed system. In the third section, an overview of the results and graphical relationships is presented. The fourth and final section presents the most important conclusions.

## 2. Experimental Methodology

In this section, the work methodology is presented, which is divided into the following:

A. Design of a circuit for measuring vital parameters and the proportion of gases in the blood.
B. Design of a circuit for measuring body resistance.
C. Merging the circuits into one device.

### 2.1. The Proposed System for Measuring Vital Parameters

**Figure 1:**
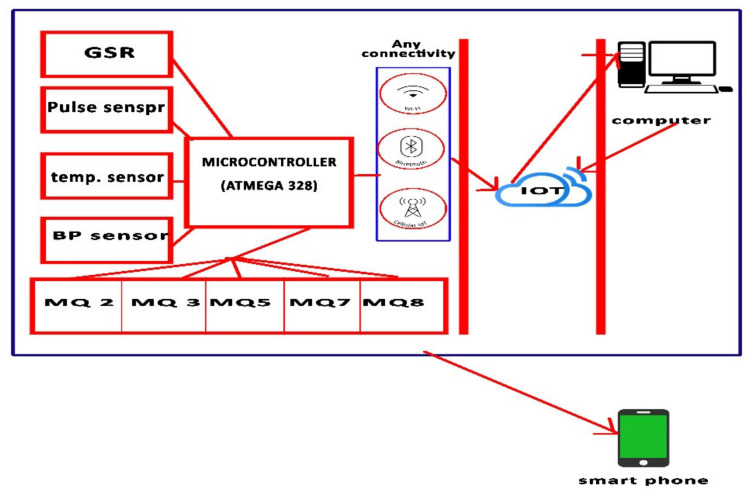
The block diagram of the design structure.

The proposed design relies on a microcontroller (ATmega328P, ATMEL, San Jose, CA, USA) as the backbone of the design, as shown in Figure 1. The proposed design is divided into two parts (measurement of vital parameters using four sensors and measurement of the level of gas in the blood using five sensors). Nine sensors were connected in this design to measure the various vital parameters to be measured. The pulse sensor was used to measure the heart rate, the pressure sensor to measure blood pressure, the temperature sensor to measure body temperature and the galvanic skin response sensor was used to measure the amount of change in the body’s resistance through the skin. Five additional sensors were also connected to the proposed system to sense different gases in the blood.

Figure 1 shows the different design stages, beginning with measuring the vital parameters of the patient or volunteer and then sending that data to the Internet of things cloud, which sends it by extension to the computer. The computer analyzes, predicts and diagnoses through a machine learning model, and then sends back the new data to the cloud to be sent to the doctor’s phone or a web interface designed to display the proposed data and diagnoses. The ATmega328P microcontroller was the backbone of the design. The sensors were connected to the noise-canceling circuit, and from there, connected to the analog-to-digital conversion circuit. The microcontroller receives the measurements and displays them on the LCD screen, and at the same time, they are sent to the cloud to be transferred to the computer using the Internet of things. A two-wire body temperature sensor was used to measure body temperature. The heart rate sensors, BMP085 and galvanic skin response were for measuring heart rate, blood pressure, body structure, and galvanic skin response, respectively. Sensors (MQ2, MQ3, MQ5, MQ7 and MQ8) were used to measure the level of gases in the blood through breathing. One of the important capabilities of the MQ sensors used is the measurement of more than one type of gas, thus the digital readings were converted to parts per million (ppm). A DAQ (data acquisition board) was connected to convert analog-to-digital values and, to overcome noise that may accompany measurements, a noise-cancellation (NC) circuit was used. The idea of noise cancellation in this paper depends on two types of inputs (basic and reference). The basic input is a primary signal that contains all the components associated with the input signal to the device; in this case, it is the measured pulse (heart rate or blood pressure), which is the basic indication. Therefore, noise cancellation is designed by choosing the filter carefully to ensure that the primary signal and the reference signal are not linked. The NC circuit was designed where the input to this circuit is the signal coming from the sensor. This signal is then inverted by changing every positive value to a negative value and every negative value to a positive value, taking into account that the amplitude and size of the signal itself do not change, and op-amps were used as an amplifier and a set of resistors and capacitors. This circuit consists of a pre-amplifier, a delay filter and a collector amplifier. The circuit’s working methodology starts with receiving the signal from the sensor, then this signal enters the pre-amplifier, which amplifies it by increasing the capacitance. Then, the delay filter delays the signal to match the received signal and enable the third stage to do its job. Finally, the amplifier collects the input (main) signal and then outputs the final signal. Sensor readings are displayed using the LCD screen. At the same time, the circuit sends the measurements in real time to the computer for analysis using MATLAB software. The proposed design in this work was designed to be portable and easy to use; thus, the presence of a group of LEDs was taken into account to clarify the patient’s condition for the user. Where the group consists of 8 LEDs, it indicates the normal, medium and critical conditions in green, yellow and red, respectively.

### 2.2. Proposed Galvanic Skin Response Circuit

**Figure 2:**
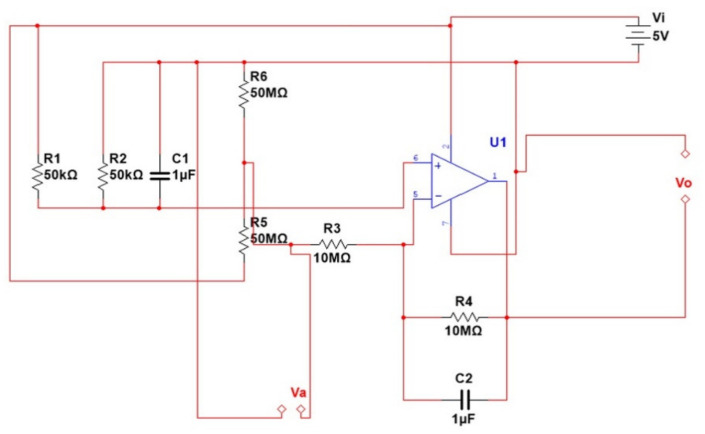
Implemented GSR circuit.

The GSR signal is used in much research and many experiments, especially regarding security services where it is used to detect lies, because the GSR signal varies according to the sympathetic nervous system of the body. By arousing human emotions, the reactions of the human body differ from one person to another. These reactions appear as changes in the vital parameters that are measured. The basic circuit working theory that we designed for the GSR is shown in Figure 2.

When there is a decrease in the resistance in the designed circuit, the voltage increases with the increase in the skin conductance of the body. Figure 2 shows that the resistance (R6) is used to measure the conduction of the skin depending on the resistance of the human body, which usually ranges from 50 kΩ to 100 MΩ. Based on the aforementioned, a resistance of 50 MΩ (R5) was used to determine the relationship between R6 and *V_o_*.

### 2.3. Merging Circuits

**Figure 3:**
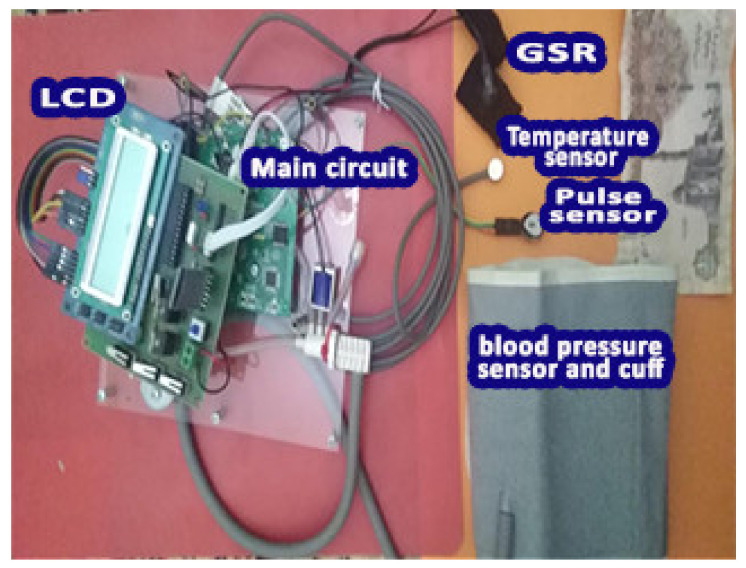
EGYXOS circuit.

Five sensors for measuring blood gas levels, as well as four sensors for measuring vital signs, were connected to the microcontroller as shown in Figure 3. The readings are converted from analog to digital and then sent to the computer for analysis; the extent of change in the parameters as they appeared are shown in the results and decision-making section.

## 3. Results

In this section, the most important results and decisions are presented, and the results are divided into several sections. First, vital indicators such as temperature, blood pressure, heart rate and body resistance were measured for a group of people (500 people). At the same time as the measurements were taken, a set of video clips was displayed, where each clip represented one of the five different emotions (anger, sadness, happiness, fear, neutrality). Special criteria were set for the test, such as: the duration of each video clip was 4 min, and all vital signs of the tested subjects were measured before, during and after watching the clips. Five sets of photos were also shown for each individual being assessed, each representing a distinct emotion. Each set of images contained 50 images depicting a particular feeling. Secondly, the results of measuring the gas levels in the blood are presented. The effectiveness of the device was tested on a group of volunteers who work continuously in places where the concentration of some gases is high, and their results were compared with normal people working in normal environments. Third, a large database was created containing the vital signs of more than 500 people, although a larger dataset will provide more insight. Fourth, a supervised machine learning model was created for prediction and diagnosis, and a set of open-source biometric data from the University of Waikato was used to train the machine learning model. WEKA 3.8.2 software was used with a biometric database configured of more than 500 people. WEKA stands for Waikato Environment for Knowledge Analysis. It is an open-source computer program developed at the University of Waikato in New Zealand. This software was used because it supports many machine learning algorithms and also has an easy-to-use interface. Among the many algorithms supported by this software, there were five machine learning algorithms with an accuracy of more than 90% to measure performance. Finally, these four sections were grouped into a program that supports IoT technology to display the results for easy understanding.

### 3.1. Effect of Emotions on Vital Parameters

Table 1 summarizes the systolic and diastolic blood pressure data obtained utilizing the microcontroller and reference device. The average results of the blood pressure tests for the five emotions of persons ranging in ages from 3 to 71 years are shown in the table below. The obtained results were compared using the developed device versus the certified reference devices from Omron and Beurer. The results of the microcontroller-based design in the sad state were similar to the findings of the reference device. In both the happy and fearful phases, represented in green and orange, the microcontroller produced results that were extremely similar to the reference device measurements.

The results shown in Table 1 indicate that the microcontroller-based design’s normal-state blood pressure readings were close to those of the reference device. The blood pressure readings in a state of anger were marked by variations that rise and fall, regardless of the fact that the microcontroller device achieved results which were almost identical to the reference device. In a state of sadness, the design involving the microcontroller produced results that were similar to those of the reference device, as illustrated in Table 1. The blood pressure readings differed in the fearful state, with the measurements of the proposed design being closer to those of the reference device. This table demonstrates that the blood pressure readings increased when people of different ages were happy; this also highlights the results of the design that employed the microcontroller, which were close to the results of the reference device.

Table 2 shows the extracted data of the pulse rate using the microcontroller and reference device. The readings in the following table show the average results of pulse rate measurements for five emotions for people ranging in age from 3 to 71 years. The results of the designed devices and Omron and Beurer devices as certified reference devices were compared. Table 2 also shows the difference between the readings of heart rate using the two devices used in the measurement, whereas the results using the device designed with a microcontroller were closest in symmetry with the results measured by the reference device. This table shows the change in the pulse rate in the normal condition, whereas the pulse rate increased in the angry condition.

Table 3 shows the temperature measurements, which ranged between 36.5 and 37 degrees Celsius and which is a slight change within the normal range, even with a change in the emotional state of people. This table shows the data extracted for the five emotions based on a body temperature sensor using a microcontroller and a reference device. Table 3 illustrates the temperature change in the normal state and the increase in the angry state. In addition, this table introduces fluctuations in temperature in the sad condition and a slight increase in temperature in the happy condition. The measurement time for the two devices was equal, as shown in Table 3.

The GSR signal is a low-frequency signal (2 Hz), thus a low-pass filter was used to remove any noise. An op-amp was used as a buffer signal to convert high impedance to low impedance. The op-amp circuit was designed according to the accepted standard design, as shown in Figure 2.

The voltage across the body resistance is:

(1) $$V_{a}=50M\Omega /\left(50M\Omega +R6\right)V_{i}\;$$

and the output voltage is:

(2) $$V_{o}=R4/R3\left(V_{ref}-V_{a}\right)+V_{ref}$$

Equation (1) shows the relationship between the voltage passing through the human body’s resistance ($V_{a}$) and the input voltage ($V_{i}$). Equation (2) shows the relationship between the output voltage ($V_{o}$) and the reference voltage ($V_{ref}$). It is responsible for preventing the op-amp from acting as an amplifier when the resistance (R6) is changed, as measured by the voltage ($V_{a}$).

**Figure 4:**
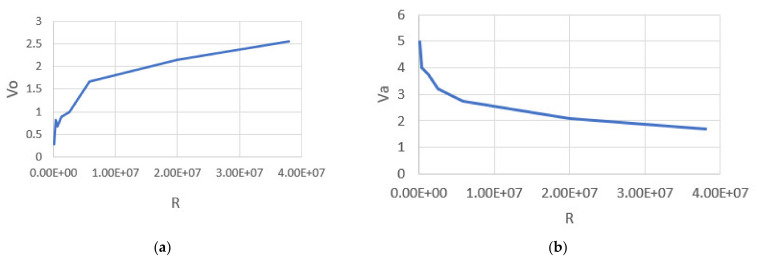
(**a**). *V_o_* vs. R based on a change in emotions. (**b**). *V_a_* vs. R based on a change in emotions.

It is noted in Table 4 that the value of R (kOhms) increases whereas the value of *V_a_* decreases. Figure 4a,b show the graphic relationship between the change in the value of *V_a_* and *V_o_* with the change in the value of the resistance R (kOhms). Figure 4a shows that the values of *V_o_* increase with increasing resistance values, and this appears clearly in the case of happiness, where the change is noticeable. Although Figure 4b is illustrative, *V_a_* values are associated with the change in resistance values and are also noticeable in the case of happiness. As the value of the resistance R increases, the value of *V_a_* increases so that the relationship here becomes an (inverse) relationship.

### 3.2. The Proportion of Some Gases in the Blood

The most important results of the measurements of gases in the blood by breathing are now presented. The MQ7, MQ2, MQ3, MQ5 and MQ8 readings are shown. Measurements were taken for a group of 100 people working in a field that exposes them to methane daily, and were compared to a group of the same number who had no or minimal exposure to methane. The same test method was conducted with the rest of the sensors to measure the percentages of different gases. As a result of the aforementioned sensors that can measure more than one gas, five types of gases were focused on. The gases that were focused on in this work are the most prevalent in usual daily work environments. The gas measurement sensor was placed inside a plastic tube with a handle to facilitate carrying so that the sensor was away from the human mouth, at a distance of no more than 7 cm. These sensors can also be used permanently for the continuous measurement of gases through the mouth, with a simple change in the design of the plastic shape to adapt to the method of use, such as by tying the tube around the head and taking into account the distance of the sensor from the mouth. The results of the MQ2 readings for methane are shown in Table 5. The table displays MQ2 readings for people exposed to daily methane and for normal people.

Table 5 illustrates the different readings of methane gas. It appears from the readings that there were many natural measurements that range between 0.811 and 5.44 ppm, which is a ratio that is closer to natural amounts. This is contrary to the readings in the right column of the table, where it shows that there were high percentages of 1003 and 776.9 ppm, which are very critical ratios. Table 6 shows the MQ3 sensor readings for daily alcohol drinkers compared to a group of the same number who did not drink or were indirectly exposed to alcohol.

Table 6 illustrates the different readings of alcohol levels. It appears from the readings that there were many natural measurements that range between 0.8 and 1.01 ppm, which is a ratio that is closer to the natural levels. From the table, it is clear that there were high levels of 328 and 498 ppm for people exposed to alcohol, which are very critical. Table 7 presents the most important readings of the MQ5 sensor for people working in the field of natural gas compared to normal people or those who are exposed to it indirectly.

People who work in an environment exposed to natural gas had measurements of 791 and 850 ppm, which are very critical levels, whereas the normal people had readings between 20 and 40 ppm, which is closer to the natural ratios; this is presented in Table 7. Table 8 presents the most important readings of the MQ7 sensor for carbon monoxide for two groups of people, one of whom is exposed on a daily basis to this gas and the other are normal people.

The measurements of normal people appeared between 50.65 and 125 ppm for the percentage of carbon monoxide in the blood, although these were large percentages for people who live in a somewhat natural environment. It also shows that the readings of people in a work environment exposed on a daily basis to carbon monoxide gas ranged between 340 and 600 ppm. Table 8 shows that these different readings are at very critical levels. Table 9 presents the most important MQ8 sensor readings for a group of people such as divers exposed on a daily basis to hydrogen gas compared to a group of normal people. The measurements of natural people were closer to normal, as they ranged between 4.7 and 8.7 ppm, whereas the table displays high levels of 50 and 70 ppm for people exposed to hydrogen gas that reach the critical state of hydrogen gas in the blood.

### 3.3. Machine Learning Model for Diagnosis and Prediction

Machine learning is a branch of computer science and is a form of artificial intelligence (AI) that teaches computers to think in a similar way as humans. Machine learning was once used to perform a particular task, but now can be programmed in such a way that it is able to learn to perform many different tasks. There are many types of machine learning, but there are two main types of machine learning that are most popular: supervised learning, which trains algorithms based on human-labeled input and output data, and unsupervised learning, which provides the algorithm with unlabeled data to allow it to search for a structure within its input data. In this work, a supervised machine learning model was used, where a set of human data regarding vital signs from the open-source medical data website (www.physionet.org) was used as input for the University of Waikato machine learning program (open source). All possible disease cases were considered that may arise according to the type of data that was entered. Then, the data and real readings taken by the proposed system were entered to make the model analyze, classify, predict and diagnose. The learning model was divided into two parts:

A. A part that categorizes and predicts the level of gases in the blood based on measurements of gas levels taken.
B. The other part analyzes, classifies and predicts (emotions and diseases) based on the measured vital parameters.

In Table 10, a set of attributes are indicated by which our machine learning model was built. This model was built to classify the initial diagnosis of people in terms of infection (influenza, allergy, COVID-19). This diagnosis is preliminary and does not dispense with the PCR procedure to confirm the infection of COVID-19. These attributes are divided into two groups, one of which indicates that the patient does not have COVID-19 disease and is indicated by the value 1. The other group is assigned values from 2 to 4, which represent the other cases of classification in terms of no disease, influenza or allergy. These attributes represented the input/output of the machine learning algorithms. Table 11 presents a group of some of the different readings and their classification according to the aforementioned values.

As can be seen from the table, the four categories (normal, rough case, very rough case, level of danger) are marked with numbers from 1 to 4, respectively, where the patient’s vital reading rates within the normal range are referred to as the normal condition. The model shows a rating of a rough case if there is a slight defect in temperature, respiratory rate or heart rate. As for the classification of a very rough case or level of danger, it is an indicator of a very high temperature and a low respiratory rate.

It is noted from the table that according to the classification of each case, action is taken in terms of informing the doctor or relatives or informing the emergency department of the nearest hospital. A rating of 1 is given if the readings are in the normal level, and therefore no action is taken. Parents and relatives are notified if the form gives a rating of 2, as this is evidence that the patient’s vital readings range between (37 °C to 38 °C, 5–69%, 40 to 59 or 120 to 129), which represents the temperature, average respiration and heart rate, respectively. The EGYXOS system sends a message to the doctor or to the emergency department of the nearest hospital when the case is classified as 3 or 4. This measure is taken because the readings show an increase in temperature, a decrease in the respiratory rate and a fluctuation in the heart rate between high and low; therefore the patient’s health condition is classified as dangerous or critical.

**Figure 5:**
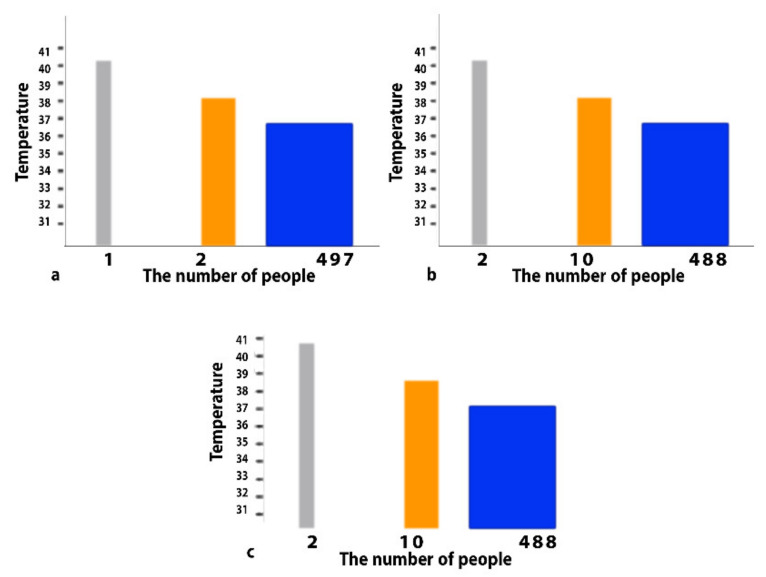
Temperature measurement analysis using MATLAB. (**a**) The temperature readings for the first time when all the people were examined. (**b**) The temperature readings for the second time when all the people were tested. (**c**) The temperature readings for the third time.

As a standard procedure for taking measurements, all test subjects’ measurements were taken every eight hours. All measurements were entered into the MATLAB software for analysis. The results showed that there were only two cases infected with COVID-19, as shown in Figure 5, and the validity of the results was confirmed by performing a PCR test for all tested groups. Figure 5a shows the temperature readings for the first time when all the people were examined. It is noteworthy that there was only one person whose temperature reached 40 degrees Celsius, which is distinguished in grey, whereas the orange color indicates the presence of two people whose temperature reached 38 degrees Celsius. Blue indicates 497 people with normal temperatures. Figure 5b shows the temperature readings for the second time when all the people were tested. In this case, two people were observed with a temperature of 40 degrees and this is indicated in grey. The orange color was intended for people with a temperature of 39 degrees Celsius, which numbered ten. The normal temperature is highlighted in blue. Figure 5c represents the temperature readings for the third time. In this case, there were only two people whose temperature exceeded 40 degrees Celsius. Orange focused on people whose temperature was 38.5 °C. The normal temperature of the tested subjects is shown in blue.

**Figure 6:**
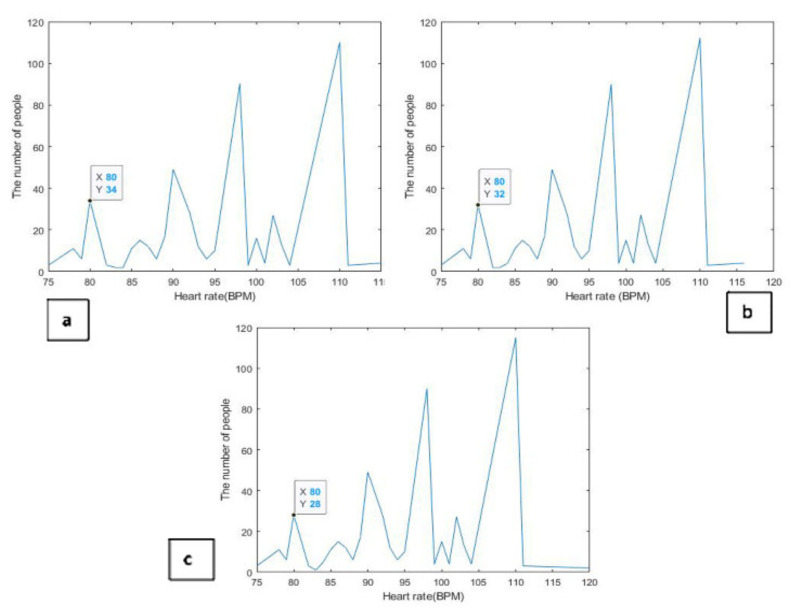
Heart rate measurement analysis using MATLAB. (**a**) The first time when the subjects were examined. (**b**) The second time of heart rate measurements. (**c**) The third time of heart rate measurements.

Similarly, at the same time as the temperature measurements were taken, heart rate measurements were taken three times with an eight hour interval in between. Figure 6a represents the readings for the first time when the subjects were examined. It appears from the graph that there was fluctuation between the upside and downside peaks, but it was within the normal range. It is noted that two cases appeared with a pulse rate that was 40 beats per minute, which is lower than normal. Figure 6b shows the second time the heart rate measurements were taken. It was observed that two subjects had a lower than normal heart rate. The measurements for the third time also showed confirmation of the presence of two cases among the group of testers whose heart rate was less than the permissible, as shown in Figure 6c. One value for the variable x and another for the variable y was taken and compared with the recorded readings to confirm the validity of all graphs.

Five algorithms were used to build a machine learning model to compare and find the best algorithm among them to help with diagnosis and predictions. The five algorithms are described in Table 12 (naive Bayes (NB), k-nearest neighbors (KNN), support vector machine (SVM), random forest (RF) and simple logistic regression (SL)), which outlines the accuracy and efficiency of each of the above-mentioned five algorithms. It is noted from the table that the SVM, random forest and simple logistic models showed accuracy rates of over 95 percent, and the SVM gave the highest percentage of each criterion (accuracy, sensitivity, specificity and F-score). However, the model based on the random forest algorithm also showed values exceeding 95 percent for each of the mentioned criteria. The model based on the simple logistic algorithm achieved higher results than expected, but its results were less than the results of the previous two models. Each of the models based on naive Bayes and KNN algorithms achieved nearly the same results and were less accurate than all other models.

The SVM, random forest, and simple logistic models show high accuracy rates, making them important models for biomedical applications for disease diagnosis and prediction. The support vector machine was the most efficient algorithm implemented in the prediction system.

### 3.4. Internet of Things Application

The proposed system (EGYXOS) was built from different technologies to realize the actual novelty of the system. The basic hierarchy of the system consists of three layers that can be described as follows:

1. LR1 (human/patient)

This layer contains the basic system (processor unit, sensors, LCD display). The human vital parameters are measured in this layer and sent to the second layer.

2. LR2 (cloud)

This is the layer responsible for providing a secure place to store data from the first layer.

3. LR3 (doctor/hospital/patient’s relatives)

**Figure 7:**
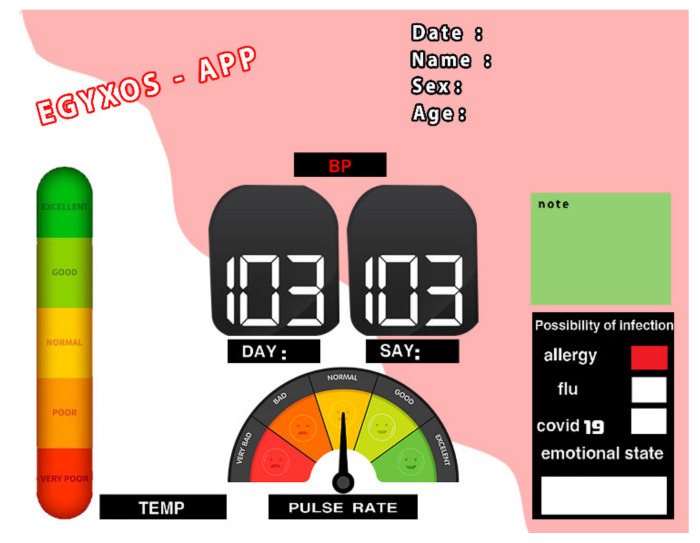
Mobile application for EGYXOS based on sensor data.

This layer receives Layer 2 data in real time to help the doctor or hospital constantly track the patient’s vital signs. In addition, the third layer also contains software specifically designed for smartphones that allows the patient’s relatives or the doctor to view the patient’s vital data in real time. Figure 7 provides an example of a portable program developed with Android Studio to display the received data in real time. As an interface for displaying received data inside hospital computers, an Internet of things web interface was designed for hospitals using Thinger.io.

Figure 7 shows the software designed for phones where the most important patient data such as age, gender, name and the latest measured vital readings are displayed. The program contains three graphic indicators that show the heart rate, respiratory rate and temperature. The graphic indicators, shown in Figure 7, consist of three basic colors: green, yellow and red. A normal status is shown in green, whereas yellow and red indicate the onset of symptoms or that the patient’s condition is critical, respectively.

### 3.5. Comparing the Proposed Work with Other Works That Support Similar Ideas

There are many studies and works on health monitoring systems using controllers and machine learning models. Despite all this, the studies referenced in the Introduction section used less data to construct their machine learning models. Additionally, some researchers resorted to using ready-made circuits such as Arduino and relied entirely on the connecting sensors without using noise-canceling circuits or a DAQ. The integrated system proposed in this paper made some major contributions in this field; one of the most important of these contributions is the creation of a huge database as a result of recording the vital readings of more than 500 people. Furthermore, this study used more than one algorithm to train the machine learning model and obtain the highest accuracy of diagnosis and prediction. Table 13 presents the differences between the previous systems and the EGYXOS system proposed in this paper.

In this work the microcontroller was used as a backbone to build the proposed system. Table 13 shows that the proposed system (EGYXOS) relied on nine sensors operating simultaneously, in contrast to the previous systems that relied on three sensors or fewer. In this study, 500 people were used to take measurements and create a large database, Whereas in previous studies, the group of test subjects ranged from 50 to 100 individuals. For the first time, the effect of human emotional states on vital parameters was presented in a manner different from previous studies. The effect of five emotions and their impact on vital parameters was analyzed, unlike in previous studies that relied on studying one or two emotions. Previous studies and systems did not address the integration of a system for detecting the proportion of gases in the blood in basic work environments with a system for measuring vital parameters. In addition to all that was mentioned, a machine learning model was added by merging more than one database of human vital parameters with the database that was created in this work to train the machine learning model. More than one algorithm was used in the machine learning model to show which one was more accurate, with the possibility of integrating more than one algorithm. The proposed system is more complex than the previous systems, as it contains many sensors and a lot of work, but an attempt has been made to remedy this by creating an application for smartphones and another for hospitals using Internet of things technology. This application and the web interface make it easier for the doctor, the patient and the hospital to easily follow the real-time status of the patient.

### 3.6. Statistical Analysis of the Proposed System Measurements

A statistical analysis technique was used to discover the accuracy between the different results taken from the reference device and the device proposed in this paper, as shown in Table 14.

From the previous tables presented in this work, it was possible to calculate the percentage of error between the results obtained using the reference device and the proposed device as shown in Table 15.

## 4. Conclusions

In this study, an integrated system based on a microcontroller and nine sensors was designed to measure critical data in real time. The proposed device was designed to rely on a large number of sensors while maintaining excellent measurement accuracy using a DAQ and noise-canceling circuits. The results measured using the proposed system were very close to the readings of the reference device. The proposed device achieved a measurement response time of 1.5 s. This work gave importance to studying the effect of human emotions on vital parameters, as the parameters increased in cases of joy and fear and decreased in the other cases. The proposed system and machine learning model designed in this paper can be used in the early diagnosis of diseases such as seventh nerve palsy and Parkinson’s disease. The proposed system has made remarkable progress to clarify the difference between the measurements of people who work in environments surrounded by gases such as carbon or hydrogen and ordinary people in normal environments. The proposed system can be used in many different applications and fields, such as military fields where this system is designed to be compatible with the helmets of pilots and marine divers and can measure the percentage of oxygen or nitrogen in the blood by breathing. It can also be used in police stations to determine the blood alcohol level of drivers, and it can also be used in emergency departments in cases of gas suffocation. This study and the models proposed in it can also be used to separate the different symptoms of a number of diseases, as the accuracy of diagnosing COVID-19 reached 64%; however, this does not exclude the need for a PCR test to confirm the infection. The system was designed with a low cost and high accuracy so that it can be used at home and in intensive care units.

## Figures and Tables

**Table 1:** Average Blood Pressure Measurements.

Age (Years Old)	Systolic BP		Diastolic BP		Time (s)		Emotion Type
	Microcontroller	Omron	Microcontroller	Omron	Microcontroller	Omron	
20	126	120	80	80	21	20	Neutral (calm)
40	120	120	80	80	25	21	
60	121	121	80	80	25	20	
20	133	131	82	85	23	22	Angry
40	155	150	94	91	24	21	
60	153	153	105	100	26	20	
20	113	113	75	75	26	20	Sad
40	95	95	65	70	24	23	
60	98	98	70	70	25	23	
20	126	120	81	81	26	21	Happy
40	120	120	79	80	24	20	
60	120	120	81	80	23	21	
20	115	115	62	60	24	20	Fearful
40	155	159	96	92	24	20	
60	139	144	90	94	24	21	

**Table 2:** Average Target Heart Rate Estimates.

Age (Years Old)	Heart Rate (Pulse Per min)		Time (s)		Emotion Type
	Microcontroller	Omron	Microcontroller	Omron	
20	75	80	21	20	Neutral (calm)
40	85	80	21	20	
60	90	79	25	21	
20	96	95	20	20	Angry
40	110	100	21	20	
60	95	99	26	20	
20	74	70	28	22	Sad
40	69	66	25	21	
60	61	64	18	20	
20	80	80	19	20	Happy
40	89	81	23	23	
60	79	79	21	20	
20	92	95	21	20	Fearful
40	99	92	21	20	
60	100	100	21	20	

**Table 3:** Average Body Temperature Measurements.

Age (Years Old)	Temperature Measurements (Degrees Celsius)		Time (s)		Emotion Type
	Microcontroller	Reference Value	Microcontroller	Reference Value	
20	36.9	36.5	60	60	Neutral (calm)
40	37	36.8	60	60	
20	37	37.5	60	60	Angry
40	37.2	37.6	60	60	
20	36.1	36.5	60	60	Sad
40	36.4	36.6	60	60	
20	37.2	37	60	60	Happy
40	36.9	36.8	60	60	
20	36.5	36.3	60	60	Fearful
40	36.5	36.2	60	60	

**Table 4:** Change in $V_{a}$ and $V_{o}$ based on emotions.

Age (Years Old)	R (kOhms)	$V_{o}$ (Volt)	$V_{a}$ (Volt)	Emotion
20	1.00E+05	0.291	4.993	neutral
60	3.80E+05	0.812	3.992	
20	6.10E+05	0.664	3.942	angry
60	1.30E+06	0.881	3.767	
20	2.60E+06	0.983	3.197	sad
60	5.80E+06	1.667	2.743	
20	2.00E+07	2.141	2.083	happy
60	3.80E+07	2.551	1.677	
20	6.60E+05	0.464	3.642	fearful
60	1.00E+06	0.581	3.467	

**Table 5:** Methane gas percentage readings between people exposed to it on a daily basis and normal people.

Normal People (ppm)	People Exposed to Methane (ppm)
5.44	1003
2.23	560.40
0.811	283.25
2.91	854
2.72	776.9

**Table 6:** Blood alcohol levels for the experimental group and normal people.

Normal People (ppm)	People Exposed to Alcohol (ppm)
0.85	498
1.00	325
0.98	315
1.01	328
0.88	300

**Table 7:** Natural gas levels for the experimental group.

Normal People (ppm)	People Exposed to Natural Gas (ppm)
40	798
39	802
29	850
20	791
25	815

**Table 8:** Carbon monoxide levels for the experimental group.

Normal People (ppm)	People Exposed to Carbon Monoxide (ppm)
100.1	600
125.02	506
89.07	340.40
90.22	390
50.65	429

**Table 9:** Hydrogen levels for the experimental group.

Normal People (ppm)	People Exposed to Hydrogen (ppm)
4.7	50
6.8	54
7.6	68
5.4	62
8.7	70

**Table 10:** Some of the input attributes used for model formation and validation.

Attribute	Description
Br	Breath rate
HR	Heart rate
TEMP	Body temperature

**Table 11:** Decision making about the patient’s state.

Temp Sensor	Breath Sensor	Pulse Sensor Per min	Action	Risk Level
<37 °C	89–98%	60 to 115	No action	1
37–38 °C	70–88%	40–59 or 116–120	Inform family	2
<38 °C	45–69%	40–59 or 120–129	Inform doctor	3
<38 °C	>45%	>40 or <130	Critical	4

**Table 12:** Performance of five machine learning algorithms.

Criterion	NB	SVM	RF	SL	KNN
Accuracy	90.40%	98.56%	95.98%	95.65	87.39
Sensitivity	89.40	98.50	96.80	96.10	87.40
F-score	88.40	98.72	96.70	96.00	86.90

**Table 13:** The differences between the EGYXOS system and the previous systems.

Elements of Comparison		This Work (EGYXOS)	[1]	[2]	[3]	[5]	[6]	[8]	[13]	[17]
Sensors		9	3	1	1 (Camera)	1	3	2	2	3
Datasets		Group of 500 people	Group of 400 people	n/a	From 200–400	12	5	n/a	n/a	n/a
DAQ circuit		yes	n/a	n/a	n/a	yes	n/a	n/a	n/a	n/a
Noise-cancellation circuit		yes	n/a	n/a	n/a	No	n/a	n/a	n/a	n/a
The number of emotions studied		5 emotions (Photos–videos)	n/a	3 emotions (Photos only)	5 emotions (Photos only)	1	n/a	n/a	n/a	n/a
Integration of more than one database of vital parameters		yes	n/a	n/a	n/a	n/a	n/a	n/a	n/a	n/a
The blood gas measurement system is integrated with the vital parameter measurement system		yes	n/a	n/a	n/a	n/a	n/a	n/a	n/a	n/a
Machine learning algorithms used		5	n/a	n/a	1	9	n/a	1	n/a	1
IoT	app	yes	n/a	n/a	n/a	n/a	n/a	n/a	n/a	n/a
	web interface	yes	n/a	n/a	n/a	n/a	yes	yes	yes	Yes

**Table 14:** The statistical analysis of the average measured data.

Parameters	Heart Rate		Blood Pressure (Diastolic)		Blood Pressure (Systolic)		Temperature	
	Reference Device	This Work (EGYXOS)	Reference Device	This Work (EGYXOS)	Reference Device	This Work (EGYXOS)	Reference Device	This Work (EGYXOS)
Min	75	75.3	80	80	75	76	77	77
Max	98	98	98	95	98	98	99	99
Mean	86.5	85.3	87.8	86.9	86	87	87.9	87.9
Median	86.5	85.9	88.5	88	86.5	86.5	88.7	88.7

**Table 15:** The percentage error between the reference device and the proposed design.

	Heart Rate	Blood Pressure (Diastolic)	Blood Pressure (Systolic)	Temperature
Percentage Error	0.9249%	1.6185%	0.911%	0.1138%

## Data Availability

Not applicable.
